# Supplementary material for: Maternal vitamin B1 is a determinant for the fate of primordial follicle formation in offspring
Source: Nat Commun. 2023 Nov 16;14:7403. doi: 10.1038/s41467-023-43261-8 (PMC10654754; doi:10.1038/s41467-023-43261-8)
Supplement: Supplementary file 1 — Supplementary Information [file 41467_2023_43261_MOESM1_ESM.pdf]

Supplementary information for:

Maternal vitamin B1 is a determinant for the fate of primordial follicle formation in offspring

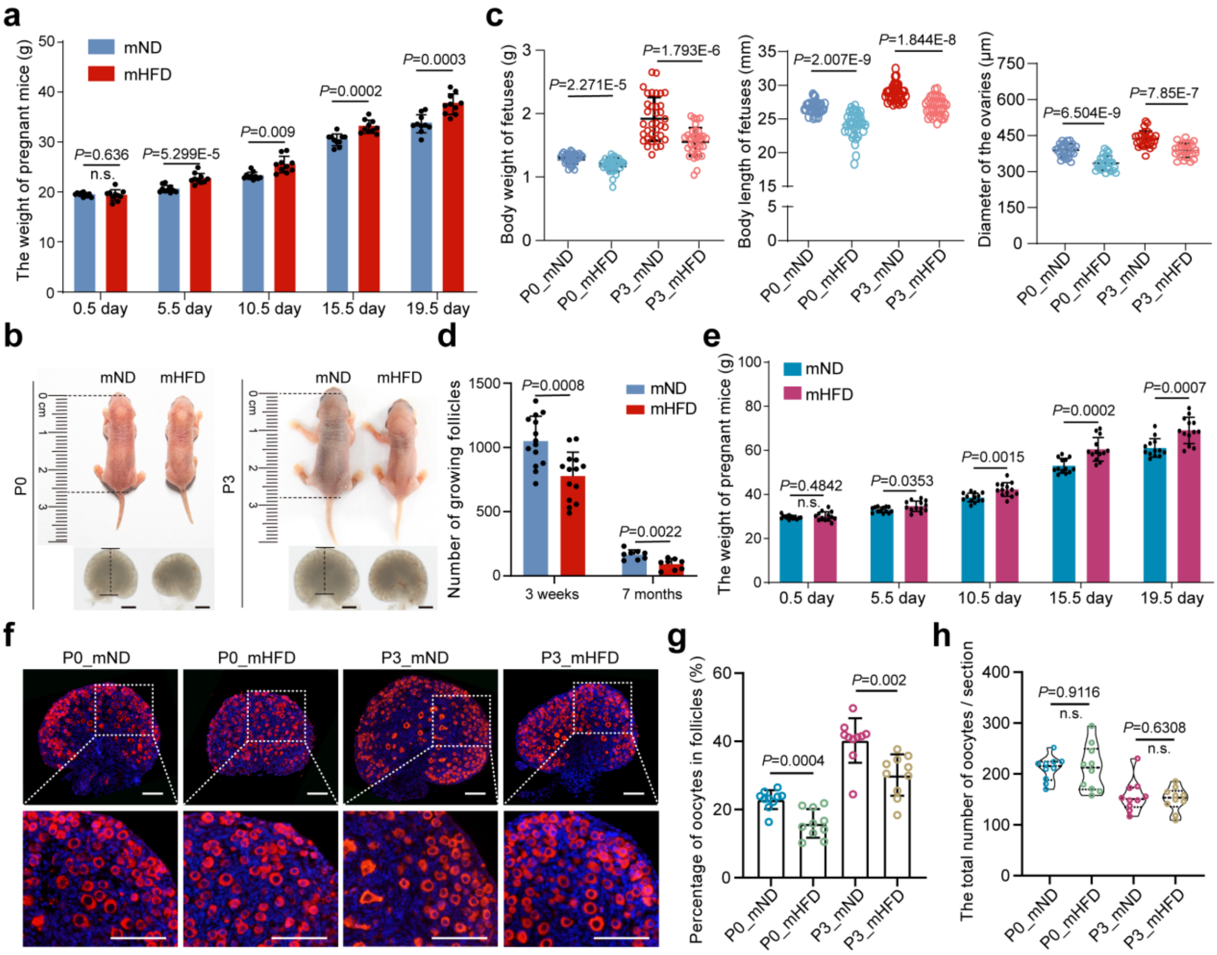

Supplementary Fig.1 Maternal HFD during pregnancy in C57BL/6 and ICR mice impaired ovarian development in offspring.

a Body weight of the ND-fed and HFD-fed pregnant C57BL/6 mice (n = 10 mice for each group). **b** Representative images showing offspring and ovary at P0 and P3. Scale bar, 100 μm. **c** Quantification of body length (n = 5 litters for each group), body weight (n = 5 litters for each group), and ovary diameter (n = 5 litters for each group) in offspring. **d** Number of growing follicles at 3 weeks (n = 14 biologically independent repeats) and 7 months (n = 8 biologically independent repeats) in offspring ovaries from mND and mHFD groups. **e** Body weight of the ND-fed and HFD-fed pregnant ICR mice (n = 13 mice for each group). **f** IF staining of DDX4 in offspring ovaries from mND and mHFD groups in ICR mice. DDX4 and DNA are stained in red and blue, respectively. DDX4 indicate oocytes. Scale bar, 100 μm. **g** The percentages of oocytes

within follicles in ICR mice (n = 10 biologically independent repeats from 10 litters for each group). **h** Violin plot showing the total number of oocytes per section in ovary in each group in ICR mice (n = 10 biologically independent repeats from 10 litters for each group). The upper and lower boundary in the plot indicates the upper and lower quantiles, the line inside the plot the median. Data were all presented as mean ± SD. A Student's t test (two-tailed) was used for statistical analysis (**a**, **c**, **d**, **e**, **g**, **h**); n.s., not significant. Source data are provided as a Source Data file.

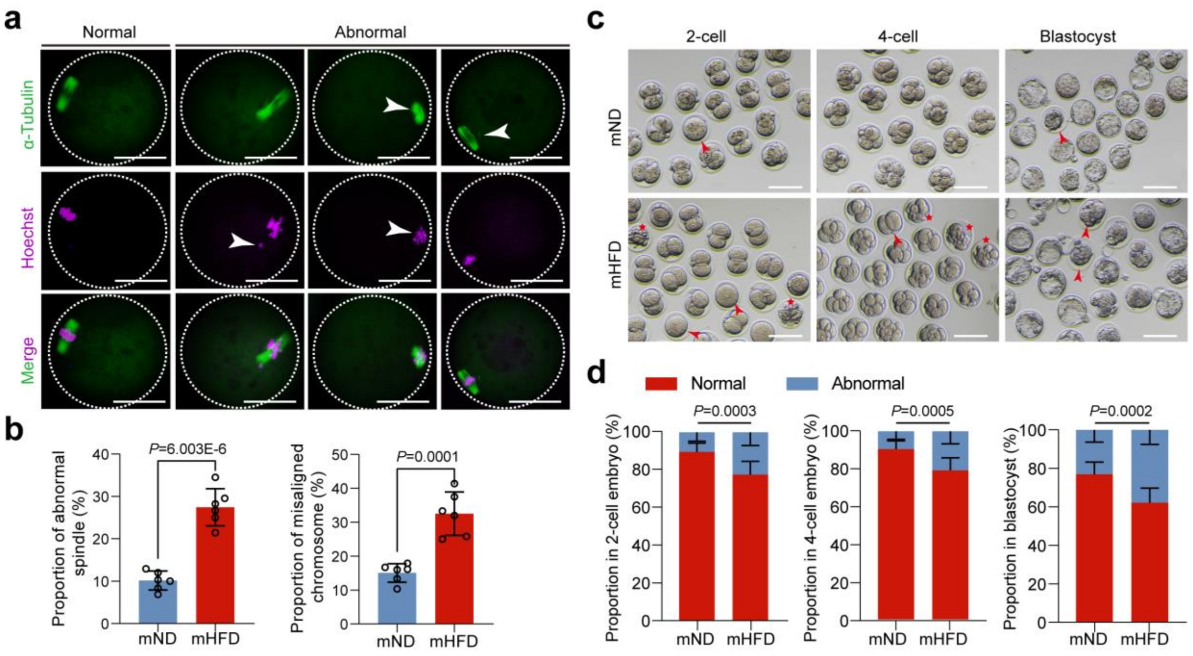

Supplementary Fig.2 Maternal HFD during pregnancy impaired oocyte quality and developmental potentiality in offspring.

a Representative images of spindle (green) morphologies and chromosome (magenta) alignment in ovulated oocytes of offspring from mND and mHFD mice. White arrows indicate aberrant spindle and misaligned chromosomes. Scale bar, 50 μm. **b** The proportion of aberrant spindles and misaligned chromosomes (n = 6 biologically independent repeats). **c** Representative bright-field images of 2-cell embryos (left), 4-cell embryos (middle) and blastocysts (right) formed by ovulated oocytes after *in vitro* fertilization from offspring of mND and mHFD mice. Red asterisks indicate examples with abnormal morphology, including cytoplasmic fragmentation; red arrows denote the abnormal embryos with developmental arrest. Scale bars, 100 μm. **d** The proportion of 2-cell embryos (left), 4-cell embryos (middle) and blastocysts (right) in each group (n = 10 biologically independent repeats). Data were all presented as mean ± SD. A Student's t test (two-tailed) was used for statistical analysis (**b**, **d**). Source data are provided as a Source Data file.

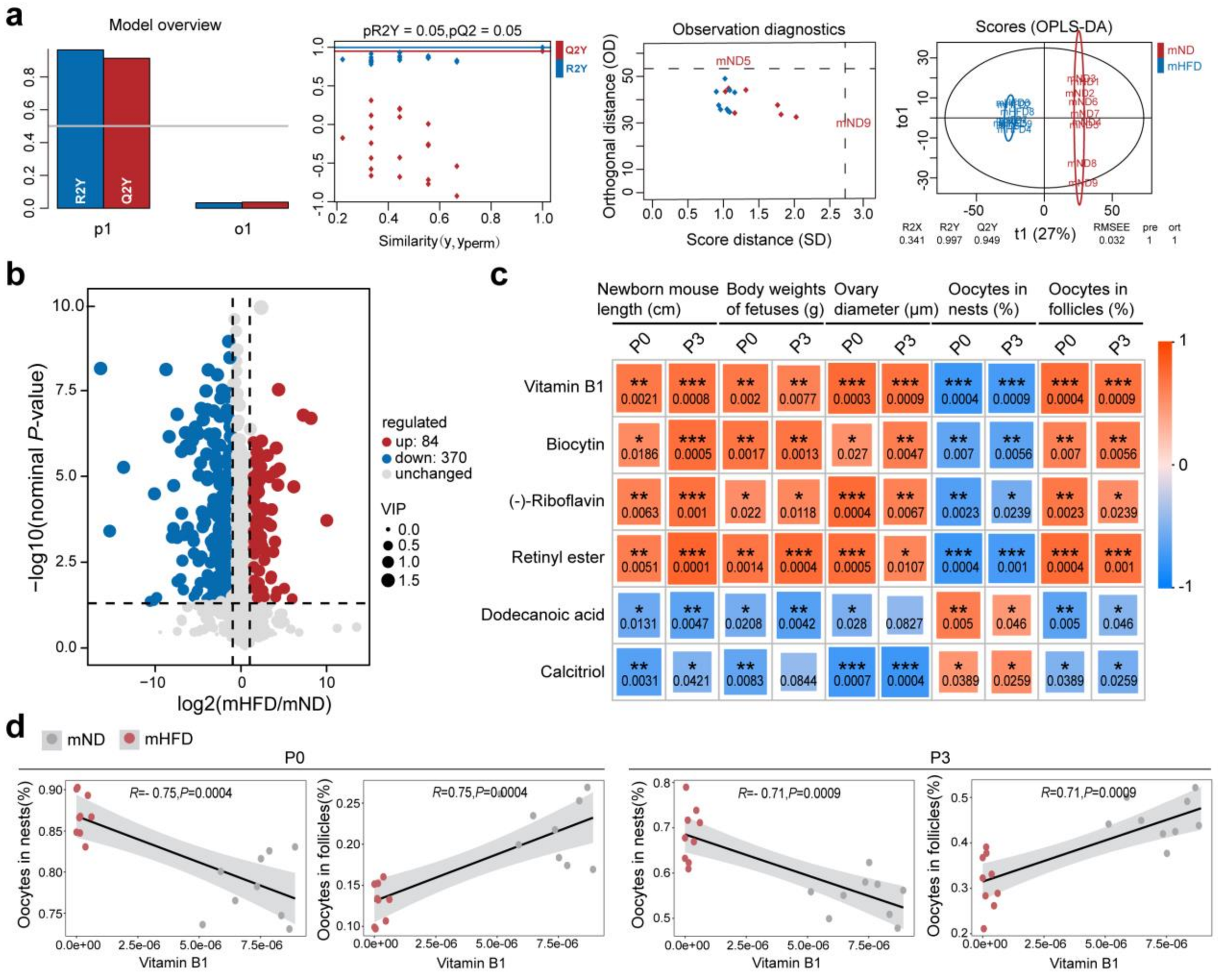

Supplementary Fig.3 Metabolomics profiling of maternal serum in mND and mHFD groups showed that the maternal vitamin B1 level was closely related to the phenotype of the female offspring.

a The OPLS-DA score plots for mND *vs.* mHFD and corresponding permutation test. **b** Volcano plots of differential metabolites in mND *vs*. mHFD comparison. **c** Spearman's correlation analysis (two-tailed) of differential metabolites levels with the phenotype of offspring. Red indicates a positive correlation, and blue indicates a negative correlation (*0.01 < *P* < 0.05, **0.001 < *P* < 0.01, ****P* < 0.001). **d** Spearman's correlation analysis (two-tailed) of vitamin B1 abundance with the proportion of oocytes within nests or within follicles. *P*-value less than 0.01 was highly significant difference. An *R*-value greater than 0 typically indicates a positive correlation between

two variables, while a value less than 0 typically indicates a negative correlation. Source data are provided as a Source Data file.

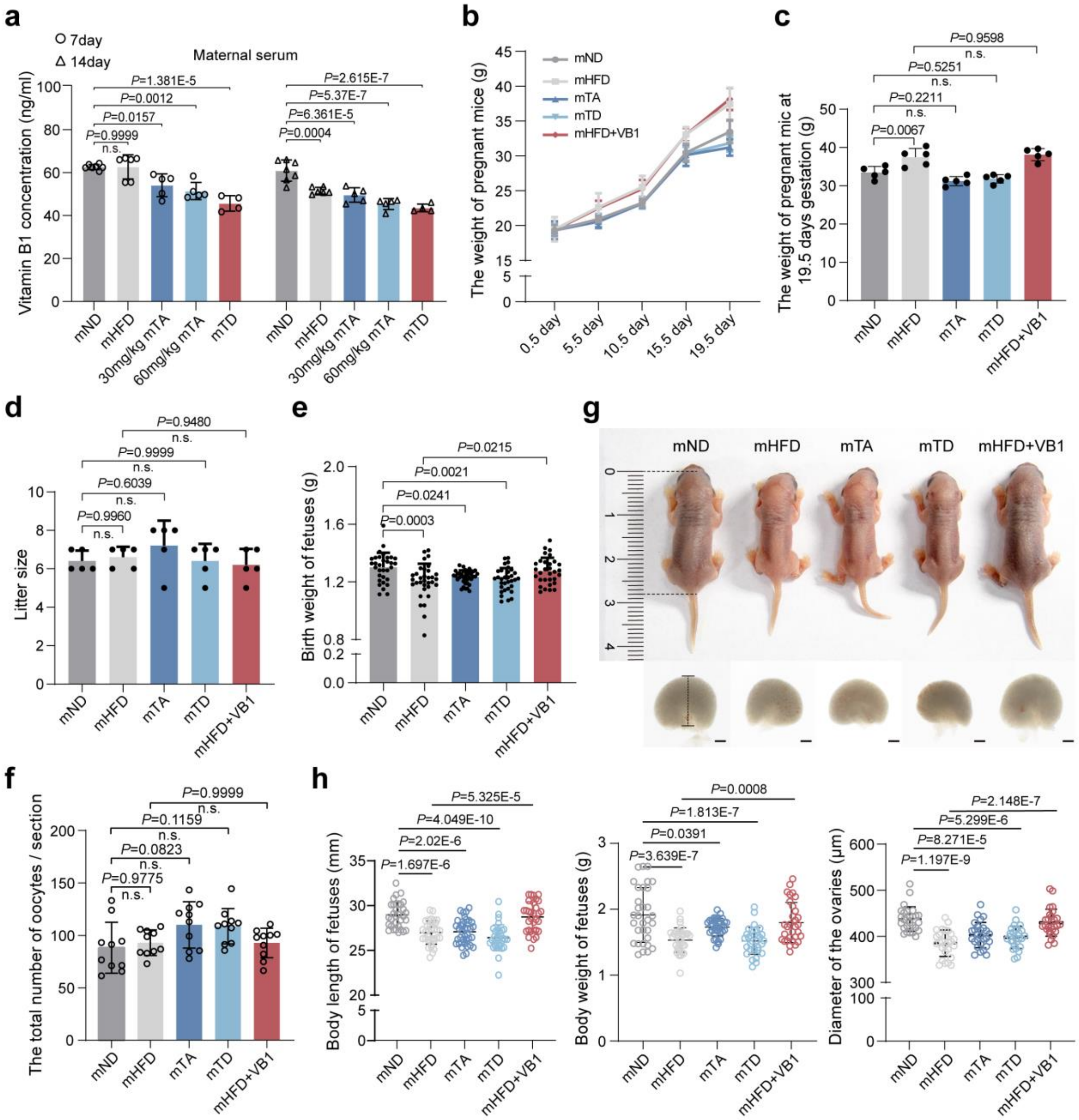

Supplementary Fig.4 Maternal vitamin B1 (VB1) levels during pregnancy exhibit a significant correlation with fetal developmental outcomes.

a The vitamin B1 concentration in serum from mND (n = 7 mice), mHFD (n = 6 mice), mTA (n = 5 mice) and mTD mice (n = 4 mice) of 7 or 14 days, respectively. **b** Body weight of pregnancy mice from mND, mHFD, mTA, mTD, and mHFD+VB1 groups (n = 5 mice for each group). **c** The weight of 19.5 days pregnant mice from mND, mHFD, mTA, mTD, and mHFD+VB1 groups (n = 5 mice for each group). **d, e** In the indicated groups, quantification of litter size and neonatal mouse

body weight (n = 5 litters for each group). **f** The total number of oocytes per section in the offspring from each group at P3 (n = 10 biologically independent repeats from 10 litters for each group). **g** Representative images of offspring and ovary in the indicated groups at P3. Scale bar, 100 μm. **h** In the indicated groups at P3, quantification of body length of fetuses (n = 5 litters for each group), body weight of fetuses (n = 5 litters for each group), and ovary diameter (n = 5 litters for each group). Data were all presented as mean ± SD. Statistical analyses were performed by one-way analysis of variance (ANOVA) with Tukey's test for multiple comparisons; n.s., not significant. Source data are provided as a Source Data file.

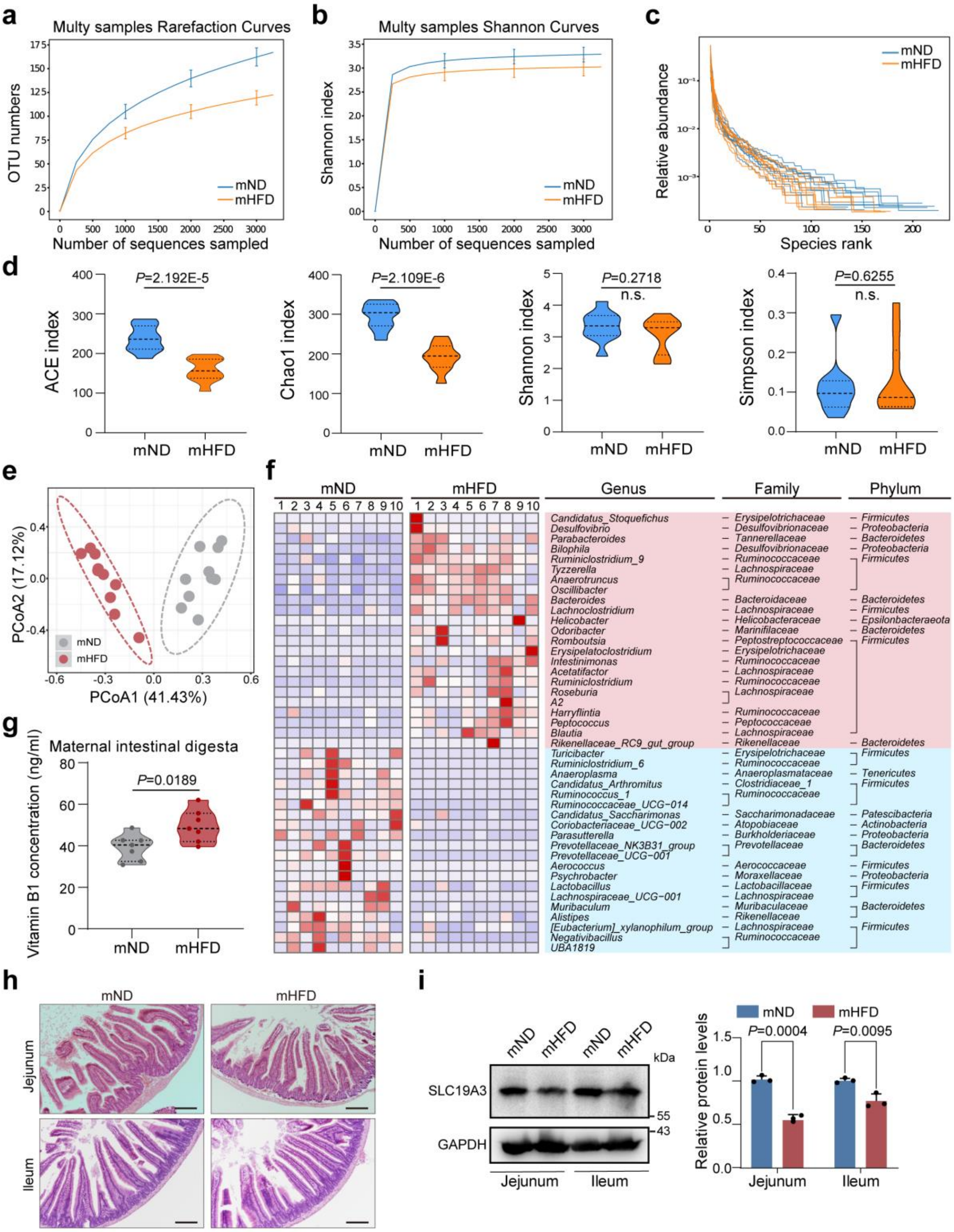

Supplementary Fig.5 Detrimental effects of HFD on pregnancy-induced intestinal microbiota and vitamin B1 absorption.

a Rarefaction curves of gut microbiota for mND and mHFD samples (n = 10 biologically independent repeats). **b** Shannon curves of gut microbiota for mND and mHFD samples (n = 10

biologically independent repeats). **c**, OTU rank curves of gut microbiota for mND and mHFD samples (n = 10 biologically independent repeats). **d** ACE index, Chao1 index, Shannon index and Simpson index of gut microbiota in mND and mHFD groups (n = 10 biologically independent repeats). **e** Bray-Curtis Principal coordinate analysis plot of gut microbiota based on the operational taxonomic unit metrics of the samples in the mND and mHFD groups (n = 10 mice for each group). **f** Heatmap of differential abundance from the mHFD group compared with the mND group (n = 10 biologically independent repeats). **g** The vitamin B1 concentration in the maternal intestinal digesta in mND and mHFD groups (n = 7 biologically independent repeats). **h** Representative H&E images of jejunum and ileum in mND and mHFD groups (n = 6 biologically independent repeats). Scale bar, 50 μm. **i** Relative protein levels of SLC19A3 of jejunum and ileum in mND and mHFD groups. GAPDH were loading controls (n = 3 biologically independent repeats). Uncropped blots in Source Data. Data were all presented as mean ± SD. A Student's t test (two-tailed) was used for statistical analysis (**d**, **g**, **i**); n.s., not significant. Source data are provided as a Source Data file.

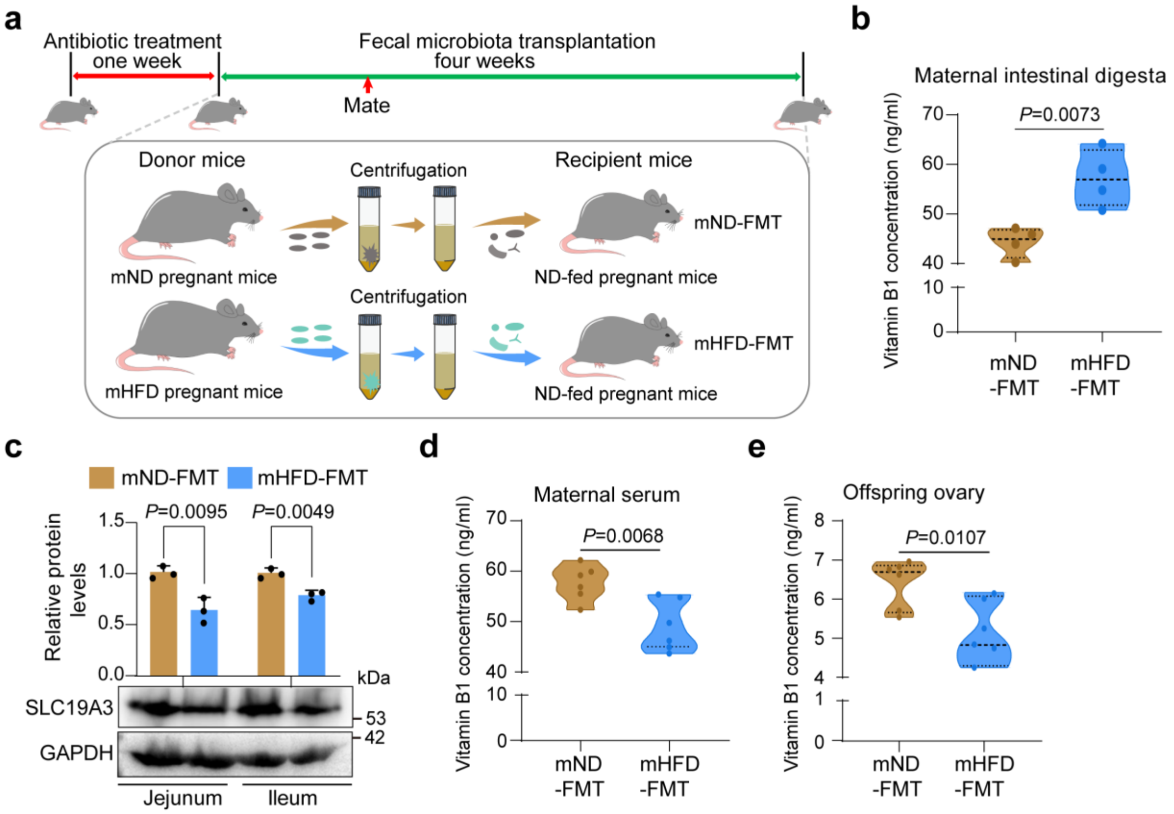

Supplementary Fig.6 Transplants of mHFD pregnant mice microbiota exhibited impaired vitamin B1 absorption.

a Study design of the fecal microbiota transplant (FMT) experiment. **b** Comparison of the vitamin B1 concentration in the maternal intestine digesta in FMT mice (n = 4 biologically independent repeats). **c** Relative protein levels of SLC19A3 of jejunum and ileum in FMT mice (n = 3 biologically independent repeats). Uncropped blots in Source Data. **d, e** The vitamin B1 concentration in maternal serum (n = 6 biologically independent repeats) and offspring ovary (n = 6 biologically independent repeats) in the indicated groups. Data were all presented as mean ± SD. A Student's t test (two-tailed) was used for statistical analysis (**b-e**). Source data are provided as a Source Data file.

a

Sample info	P0_mND	P0_mHFD	P3_mND	P3_mHFD
Estimated Number of Cells	6000	6000	6000	6000
Valid Barcodes	97.4%	98.1%	97.5%	98.0%
Mean Reads per Cell	101,135	86,927	93,851	84,151
Median Genes per Cell	2,844	3,898	2,768	4,000
Total Genes Detected	22,346	22,912	21,881	22,356
Reads Mapped to Genome	92.4%	94.2%	93.4%	94.9%
Reads Mapped Confidently to Transcriptome	66.6%	62.0%	67.2%	60.1%

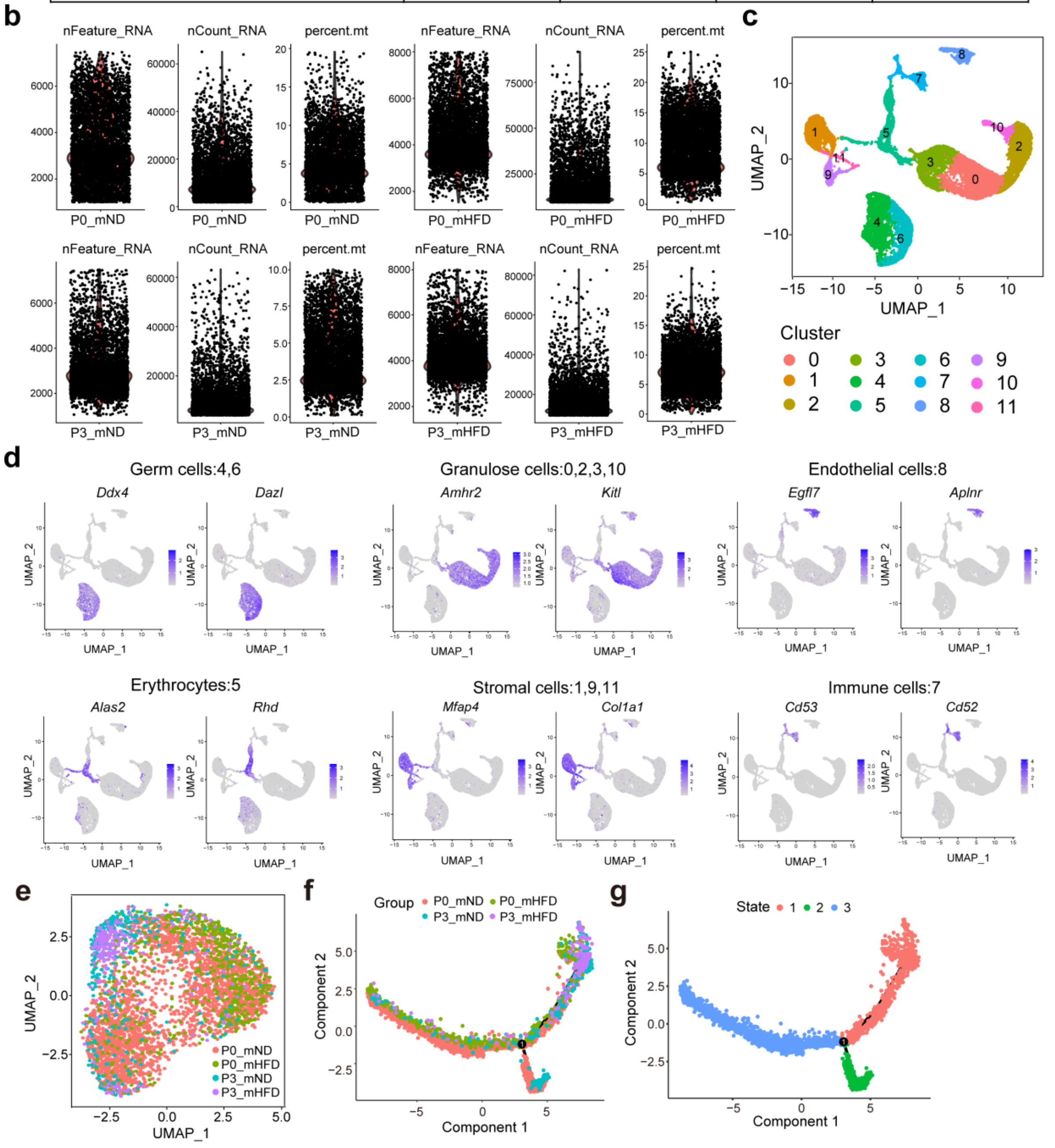

Supplementary Fig.7 Ovarian single-cell RNA sequencing information and cell clusters

identification.

a The sequenced information of four samples. **b** Violin plot demonstrating the number of genes (nFeature_RNA), unique molecular identifier (nCount_RNA) and percentage of mitochondria genes (percent.mt) in the different data sets. **c** UMAP plot labelled with cell identities. **d** The UMAP map of the expression level of cell marker genes in each type. Germ cells: *Ddx4*, *Dazl*; Granulosa cells: *Amhr2*, *Kitl*; Stromal cells: *Mfap4*, *Colla1*; Endothelial cells: *Egfl7*, *Aplnr*; Erythrocytes: *Alas2*, *Rhd*; Immune cells: *Cd53*, *Cd52*. **e** UMAP diagram of germ cell populations. **f** Single-cell trajectories of germ cell subsets. **g** Single-cell trajectories of the three germ cell states. Source data are provided as a Source Data file.

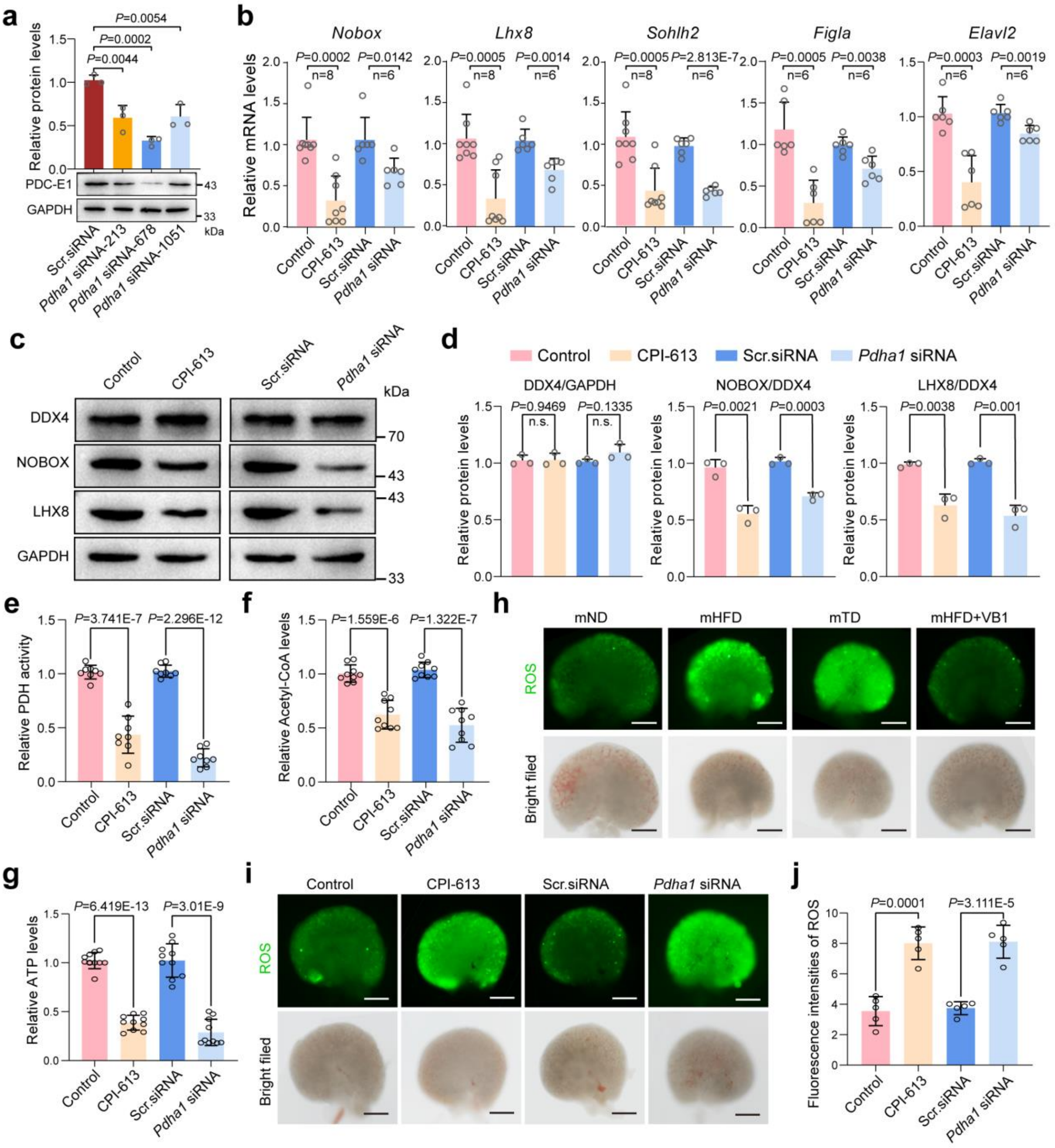

Supplementary Fig.8 Inhibition of PDH function in ovarian cells affects oocyte specific gene expression and mitochondrial function.

a Relative protein levels of PDC-E1 after ovarian transfection with *Pdha1*-siRNA for 6-h and cultured for 72-h. GAPDH were loading controls (n = 3 biologically independent repeats). Uncropped blots in Source Data. **b** RT-qPCR analyzing the expression for *Nobox*, *Lhx8*, *Sohlh2*, *Figla* and *Elavl2* in ovaries treated with CPI-613, scrambled-siRNA (Scr.siRNA) or *Pdha1*-siRNA,

respectively. The number of biologically independent repeats is indicated (n). **c**, **d** Representative images and relative protein levels of DDX4, NOBOX and LHX8 in ovaries treated with CPI-613, Scr.siRNA or *Pdha1*-siRNA, respectively (n = 3 biologically independent repeats). Uncropped blots in Source Data. **e, f** Relative PDH activity (n = 8 biologically independent repeats) and relative Acetyl-CoA levels (n = 9 biologically independent repeats) in ovaries treated with CPI-613, Scr-siRNA or *Pdha1*-siRNA, respectively. **g** Relative ATP levels in ovaries treated with CPI-613, Scr.siRNA or *Pdha1*-siRNA, respectively (n = 10 biologically independent repeats). **h** Representative image of ROS in offspring ovaries from mND, mHFD, mTD or mHFD+VB1 mice, respectively. Scale bar, 100 μm. **i**, **j** Representative images and fluorescence intensity of ROS in ovaries treated with CPI-613, Scr.siRNA or *Pdha1*-siRNA, respectively (n = 5 biologically independent repeats). Scale bar, 100 μm. Data were all presented as mean ± SD. Statistical analyses were performed by one-way analysis of variance (ANOVA) with Tukey's test for multiple comparisons (**a**) or two-tailed student's t test (**b**, **d-f**, **g**, **j**); n.s., not significant. Source data are provided as a Source Data file.

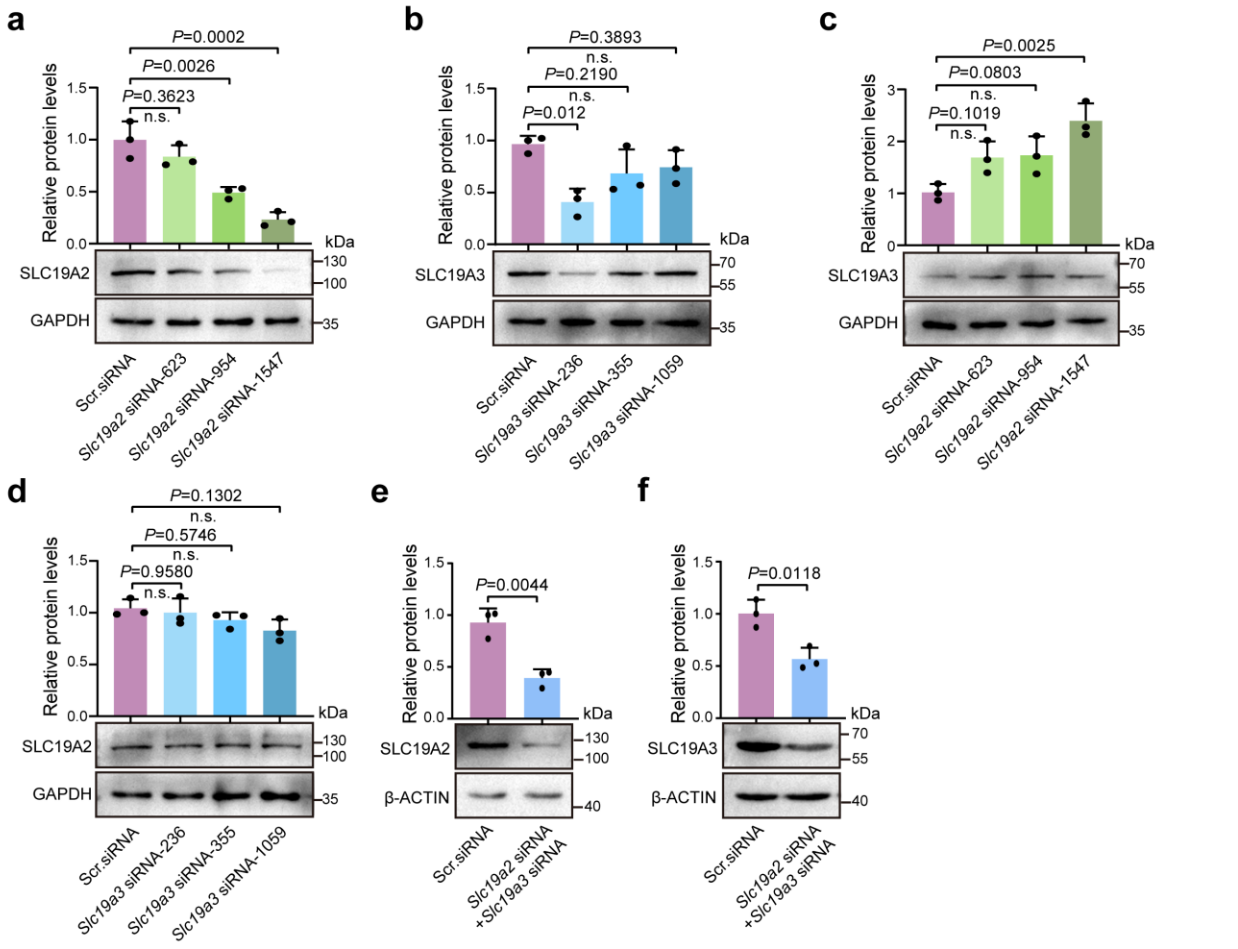

Supplementary Fig.9 Optimization of SLC19A2 and SLC19A3 for interference efficiency screening.

a Relative protein levels of SLC19A2 after ovarian transfected with *Slc19a2*-siRNA for 6-h and cultured for 72-h. GAPDH were loading controls (n = 3 biologically independent repeats). Uncropped blots in Source Data. **b** Relative protein levels of SLC19A3 after ovarian transfected with *Slc19a3*-siRNA for 6-h and cultured for 72-h. GAPDH were loading controls (n = 3 biologically independent repeats). Uncropped blots in Source Data. **c** Relative protein levels of SLC19A3 after ovarian transfected with *Slc19a2*-siRNA for 6-h and cultured for 72-h. GAPDH were loading controls (n = 3 biologically independent repeats). Uncropped blots in Source Data. **d** Relative protein levels of SLC19A2 after ovarian transfected with *Slc19a3*-siRNA for 6-h and cultured for 72-h. GAPDH were loading controls (n = 3 biologically independent repeats).

Uncropped blots in Source Data. **e** Relative protein levels of SLC19A2 in ovaries treated with Scr-siRNA or *Slc19a2*-siRNA+*Slc19a3*-siRNA, respectively (n = 3 biologically independent repeats). Uncropped blots in Source Data. **f** Relative protein levels of SLC19A3 in ovaries treated with Scr-siRNA or *Slc19a2*-siRNA+*Slc19a3*-siRNA, respectively (n = 3 biologically independent repeats). Uncropped blots in Source Data. Data were all presented as mean ± SD. Statistical analyses were performed by one-way analysis of variance (ANOVA) with Tukey's test for multiple comparisons (**a-d**) or two-tailed student's t test (**e**, **f**); n.s., not significant. Source data are provided as a Source Data file.

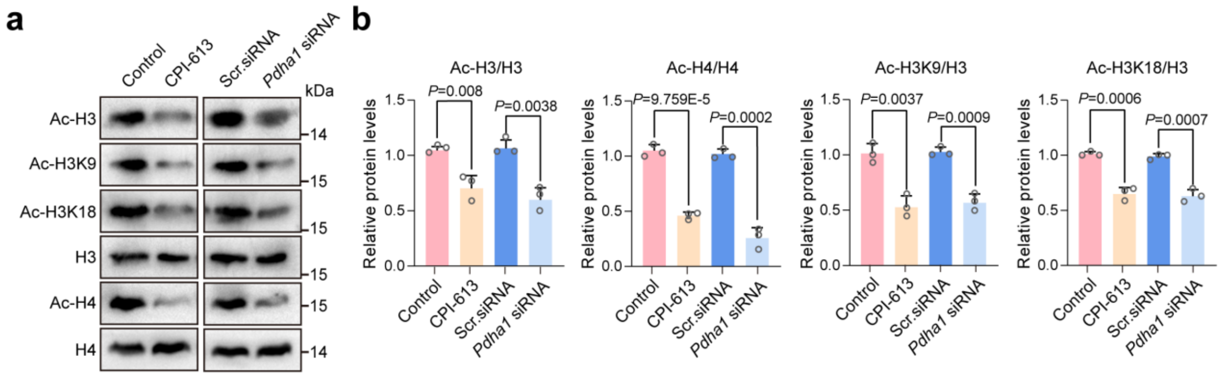

Supplementary Fig.10 Inhibition of PDH activity affects ovarian histone acetylation.

Representative image (**a**) and relative protein levels (**b**) of Ac-H3, Ac-H3K9, Ac-H3K18 and Ac-H4 in ovaries treated with CPI-613, Scr.siRNA or *Pdha1*-siRNA, respectively. H3 and H4 were loading controls (n = 3 biologically independent repeats). Uncropped blots in Source Data. Data were all presented as mean ± SD. The two-tailed student's t test was used for statistical analysis. Source data are provided as a Source Data file.

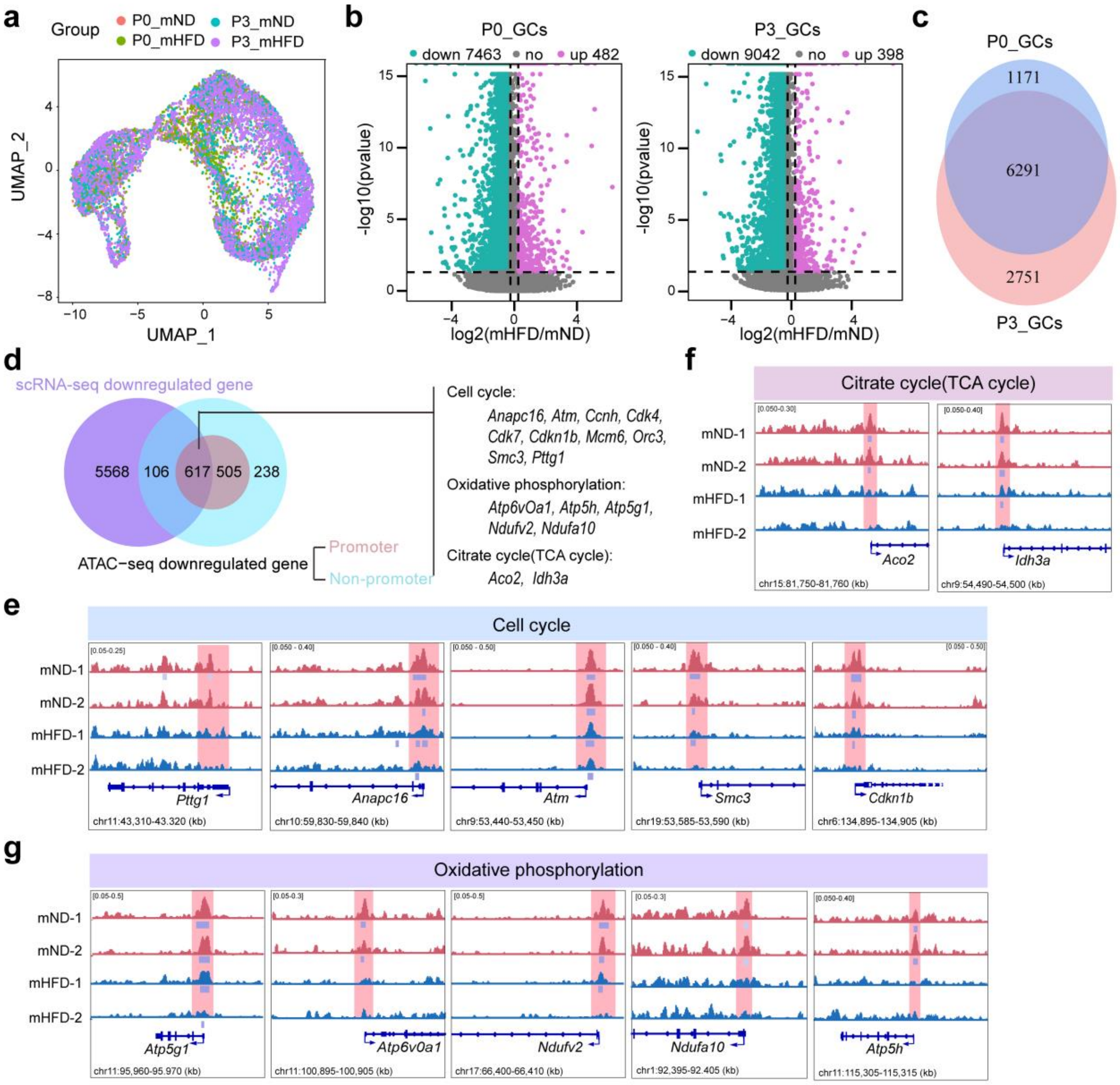

Supplementary Fig.11 The gene expression analysis of ovarian granulosa cells by ATAC-sequencing and scRNA-sequencing.

a Clustering of granulosa cells based on UMAP colored by the sample groups. **b** Volcano plot demonstrating differentially expressed genes (DEGs) in granulosa cells at P0 and P3 from offspring of mND and mHFD mice. **c** Venn diagram illustrating the common downregulated DEGs between P0_GCs and P3_GCs. **d** Venn diagram illustrating the common downregulated DEGs between ATAC-sequencing and scRNA-sequencing (left); the genes list of the cell cycle, oxidative phosphorylation and citrate cycle (TCA cycle) pathway (right). **e** ATAC-sequencing normalized

reads shown for cell cycle genes (*Pttg1*, *Anapc16*, *Atm*, *Smc3* and *Cdkn1b*) (n = 2 biologically independent repeats). **f** ATAC-sequencing normalized reads shown for TCA cycle genes (*Aco2* and *Idh3a*) (n = 2 biologically independent repeats). **g** ATAC-sequencing normalized reads shown for oxidative phosphorylation genes (*Atp5g1*, *Atp6vOa1*, *Ndufv2*, *Ndufa10* and *Atp5h*) (n = 2 biologically independent repeats). Source data are provided as a Source Data file.

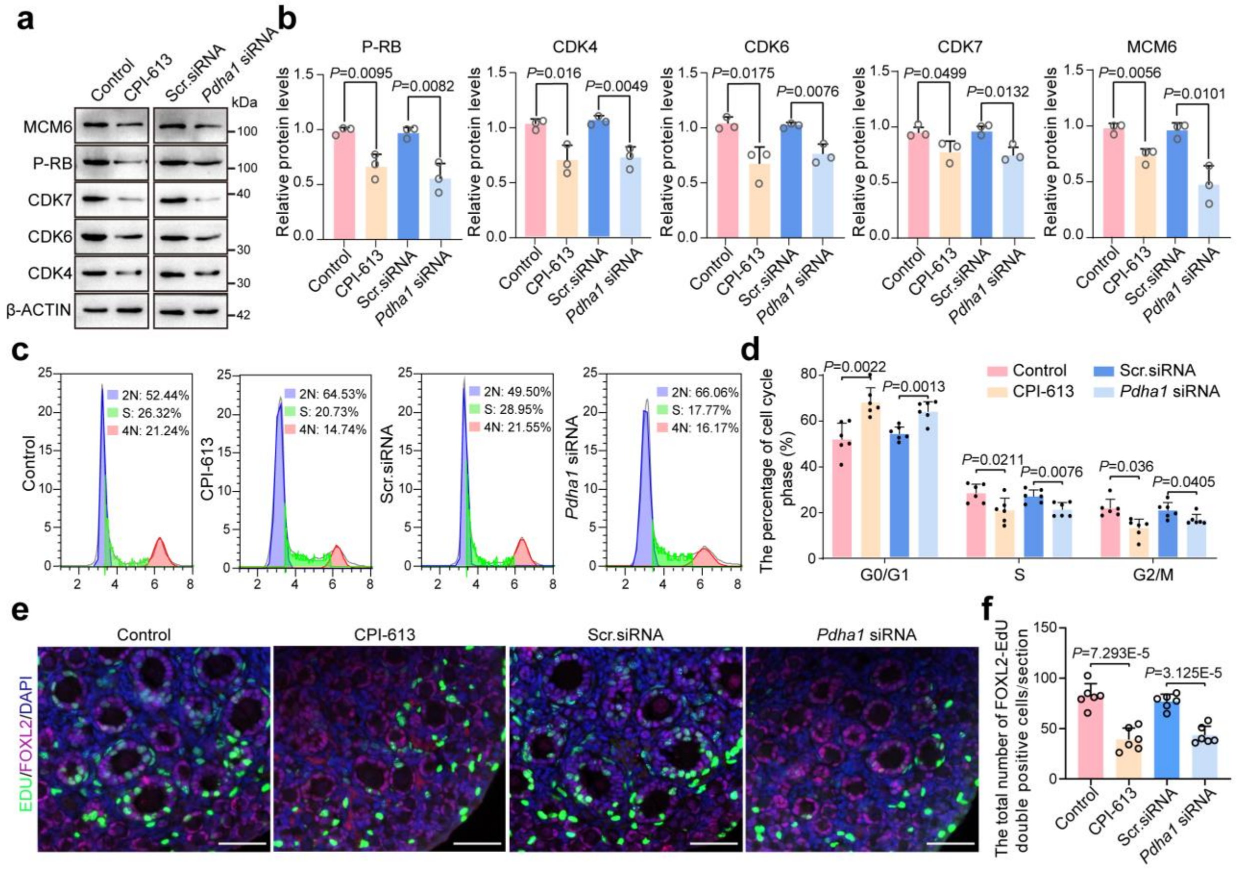

Supplementary Fig.12 Inhibition of PDH activity affects granulosa cells proliferation in offspring.

a, **b** Representative images and relative protein levels of MCM6, P-RB, CDK7, CDK4 and CDK6 in ovaries treated with CPI-613, Scr.siRNA or *Pdha1*-siRNA, respectively (n = 3 biologically independent repeats). β-ACTIN were loading controls. Uncropped blots in Source Data. **c**, **d** Representative image and the distribution of cell cycle phase in ovarian cells treated with CPI-613, Scr.siRNA or *Pdha1*-siRNA, respectively (n = 6 biologically independent repeats). **e**, **f** IF staining of EdU (Proliferation labeling) and FOXL2 (Granular cell marker), and quantification of FOXL2-EdU double-positive cells ovarian section following treatment with CPI-613, Scr.siRNA or *Pdha1*-siRNA, respectively (n = 6 biologically independent repeats). EdU, FOXL2, and DNA are stained in green, magenta, and blue, respectively. Scale bar, 50 μm. Data were all presented as mean ± SD. Student's t test (two-tailed) was used for statistical analysis (**b**, **d**, **f**). Source data are provided as

a Source Data file.

Supplementary Table 1. Characteristics of study participants.

Characteristics	HC (n = 31)	GDM (n = 31)	P-value
Age (years)	30.65 ± 3.82	32.77 ± 3.56	0.0267
Smoking status	none	none	—
Drinking status	none	none	—
Family history of diabetes	none	none	—
Peripartum antibiotics	none	none	—
Participants height (cm)	162.5 ± 4.69	161.7 ± 5.61	0.52 (n.s.)
Pre-pregnant women weight (kg)	57.32 ± 4.96	58.81 ± 8.77	0.42 (n.s.)
Pre-pregnant women BMI (kg/m^2)	21.70 ± 1.60	22.43 ± 2.67	0.20 (n.s.)
Fasting plasma glucose (mmol/L)	4.10 ± 0.32	4.34 ± 0.43	0.0072
1-hour post-load plasma glucose (75-g oGTT, mmol/L)	7.19 ± 1.42	10.25 ± 0.84	6.353E-15
2-hour post-load plasma glucose (75-g oGTT, mmol/L)	6.52 ± 0.85	9.20 ± 1.47	2.193E-12
Blood pressure (systolic pressure / diastolic blood pressure)	112 ± 11.49 / 61.61 ± 7.21	117.5 ± 11.15 / 65.45 ± 10.66	0.06 (n.s.) / 0.10 (n.s.)
Pregnant women weight (kg)	63.18 ± 6.55	67.25 ± 12.35	0.11 (n.s.)
Pregnant women BMI (kg/m^2)	23.9 ± 2.05	25.59 ± 3.65	0.028

HC, Healthy controls; GDM, Gestational diabetes mellitus; BMI, Body mass index. In this table, the data represent the mean ± SD. Statistical analyses were performed by two-tailed Student's *t*-test; n.s., not significant.

Supplementary Table 2. Antibodies used in this paper.

Primary antibodies	Vendor	Dilution	Source
DDX4 (IF/WB/IHC)	Abcam (ab13840)	1:200/1:1000/1:200	Rabbit
DDX4 (IF)	Abcam (ab27591)	1:200	Mouse
LHX8 (IHC/WB)	Abcam (ab137036)	1:150/1:800	Rabbit
NOBOX (IHC/WB)	SANTA (sc-514178)	1:150/1:1000	Mouse
α-tubulin (IF/WB)	CST (3873)	1:1000/1:1000	Mouse
SLC19A3 (WB)	Proteintech (13407-1-AP)	1:1000	Rabbit
GAPDH (WB)	Proteintech (60004-1-IG)	1:1000	Mouse
β-Actin (WB)	Sigma (A1974)	1:1000	Mouse
PDH-E1 (WB/IF)	CST (3205)	1:1000/1:200	Rabbit
H3 (WB)	Proteintech (17168-1-AP)	1:1000	Rabbit
Ac-H3 (WB)	Affinity (AF4365)	1:1000	Rabbit
Ac-H3K9 (WB)	CST (9649)	1:1000	Rabbit
Ac-H3K18 (WB)	CST (13998)	1:1000	Rabbit
H4 (WB)	Proteintech (16047-1-AP)	1:1000	Rabbit
Ac-H4 (WB)	ActiveMotif (39026)	1:1000	Rabbit
P-RB (WB)	CST (8516)	1:1000	Rabbit
CDK4 (WB)	Proteintech (11026-1-AP)	1:1000	Rabbit
CDK6 (WB)	Proteintech (14052-1-AP)	1:1000	Rabbit
MCM6 (WB)	Affinity (DF6716)	1:1000	Rabbit
CDK7 (WB)	Affinity (DF6559)	1:1000	Rabbit
FOXL2 (IF)	Abcam (ab5096)	1:60	Goat
**Secondary antibodies**			
CyTM-3-conjugated donkey anti-rabbit (IF)	Jackson (711-165-152)	1:200	Donkey
FITC-conjugated donkey anti-rabbit (IF)	Jackson (711-095-152)	1:200	Donkey
FITC-conjugated donkey anti-mouse (IF)	Jackson (715-095-150)	1:200	Donkey
HRP-conjugated goat anti-Mouse IgG (IHC/WB)	Thermofisher (31430)	1:100/1:1000	Goat
HRP-conjugated goat anti-rabbit IgG (IHC/WB)	Thermofisher (31460)	1:100/1:1000	Goat
Cy3-labeled donkey anti-Goat IgG (IF)	Beyotime (A0502)	1:100	Donkey

Supplementary Table 3. Primers Used for Quantitative RT-PCR.

Genes	Genbank	Forward primer sequence	Reverse primer sequence
Lhx8	NC_000069.5	CAGTTCGCTCAGGACAACAA	CCTGCAGTTCTGAAACCACA
Figla	NM_012013	CCGCCATCTGTAGGCTCAAG	ACACAGCCGAGTATCTGTATGTA
Sohlh2	NM_028937.3	TCTCAGCCACATCACAGAGG	GGGGACGCGAGTCTTATACA
Nobox	NM_130869	CTATCCTGACAGTGACAAACGCC	CACCCTCTCAGCACCCTCATTAT
Elavl2	NM_207685	ACACAGCCAATGGTCCAACC	TTCCCGGAGTCAACTGGTGA
Ddx4	NM_010029	GCTTCATCAGATATTGGCGAGT	GCTTGGAAAACCCTCTGCTT
Gapdh	NC_010447.5	AGGTCGGTGTGAACGGATTTG	TGTAGACCATGTAGTTGAGGTCA
β-actin	NM_007393	GGCTGTATTCCCCTCCATCG	CCAGTTGGTAACAATGCCATGT
Ccnd3	NM_001081635	CGAGCCTCCTACTTCCAGTG	GGACAGGTAGCGATCCAGGT
Cdk4	NM_009870	ATGGCTGCCACTCGATATGAA	TCCTCCATTAGGAACTCTCACAC
Cdk6	NM_009873	GGCGTACCCACAGAAACCATA	AGGTAAGGGCCATCTGAAAACT
Cdk7	NM_009874	TACACACATCAAGTTGTGACCAG	CCAGGATACGGAGAAAAACCAG
Mcm6	NM_008567	GCTGTTCCTAGACTTCCTGGA	CAACCAGCGTGTTTCTCTCAG
Orc3	NM_015824	CACCTCCTCCGTGTCTAAGG	TCATCCGTTGCCATAGTAAGCTA
Amhr2	NM_144547	GGGGCTTTGGACACTGCTT	GTCTCGGCATCCTTGCATCTC
Dnajc19	NM_001026211	GCACAGTGGTAGCAGTCGG	CATGCTTCATGGCTTGTAAAACA
Timm17a	NM_011590	TGCCCCTGGCGAATTGTAG	CTGTCAAACTTCCTCGGAGTC
Tomm20	NM_024214	GCCCTCTTCATCGGGTACTG	ACCAAGCTGTATCTCTTCAAGGA
Uqcr10	NM_197979	ATCCCTTCGCGCCTGTACT	GTGCTCGTAGATCGCGTCT
Ndufa2	NM_010885	TTGCGTGAGATTCGCGTTCA	ATTCGCGGATCAGAATGGGC
Ndufa3	NM_025348	ATGGCCGGGAGAATCTCTG	AGGGGCTAATCATGGGCATAAT

Supplementary Table 4. The sequence Used for siRNA.

Genes	sense	antisense
Gapdh	CACUCAAGAUUGUCAGCAATT	UUGCUGACAAUCUUGAGUGAG
Pdha1-213	GAUGCUACAUUUGAGAUUATT	UAAUCUCAAAUGUAGCAUCTT
Pdha1-678	GAGGUCUGUUUGACAUUAUTT	AUAAUGUCAAACAGACCUCTT
Pdha1-1051	GUAAGAGUGACCCUAUUAUTT	AUAAUAGGGUCACUCUUACTT
Scr.siRNA	UUCUCCGAACGUGUCACGUTT	ACGUGACACGUUCGGAGAATT
Slc19a2-623	GCCUACUACUCCUAUAUCUTT	AGAUAUAGGAGUAGUAGGCTT
Slc19a2-954	GGGAGGACAUUGAGUCAAATT	UUUGACUCAAUGUCCUCCCTT
Slc19a2-1547	GGCCUUGGCUUAUGUAUCATT	UGAUACAUAAGCCAAGGCCTT
Slc19a3-236	GAGCAACUCUUGGGUUUAUTT	AUAAACCCAAGAGUUGCUCTT
Slc19a3-355	GCCCAGAGAUGACAAAUGATT	UCAUUUGUCAUCUCUGGGCTT
Slc19a3-1059	GCAGGUUAUAACCAAAUCUTT	AGAUUUGGUUAUAACCUGCTT
