# Supplementary material for: Maternal vitamin B1 is a determinant for the fate of primordial follicle formation in offspring
Source: Nat Commun. 2023 Nov 16;14:7403. doi: 10.1038/s41467-023-43261-8 (PMC10654754; doi:10.1038/s41467-023-43261-8)
Supplement: Supplementary file 3 — Reporting Summary [file 41467_2023_43261_MOESM3_ESM.pdf]

nature portfolio

Corresponding author(s): Yang Zhou, Teng Zhang

Last updated by author(s): 2023-10-21

Reporting Summary

Nature Portfolio wishes to improve the reproducibility of the work that we publish. This form provides structure for consistency and transparency in reporting. For further information on Nature Portfolio policies, see our Editorial Policies and the Editorial Policy Checklist.

Please do not complete any field with "not applicable" or n/a. Refer to the help text for what text to use if an item is not relevant to your study.
For final submission: please carefully check your responses for accuracy; you will not be able to make changes later.

Statistics

For all statistical analyses, confirm that the following items are present in the figure legend, table legend, main text, or Methods section.

n/a	Confirmed	
☐	√	The exact sample size (n) for each experimental group/condition, given as a discrete number and unit of measurement
☐	√	A statement on whether measurements were taken from distinct samples or whether the same sample was measured repeatedly
☐	√	The statistical test(s) used AND whether they are one- or two-sided<br>*Only common tests should be described solely by name; describe more complex techniques in the Methods section.*
√	☐	A description of all covariates tested
☐	√	A description of any assumptions or corrections, such as tests of normality and adjustment for multiple comparisons
☐	√	A full description of the statistical parameters including central tendency (e.g. means) or other basic estimates (e.g. regression coefficient) AND variation (e.g. standard deviation) or associated estimates of uncertainty (e.g. confidence intervals)
☐	√	For null hypothesis testing, the test statistic (e.g. F, t, r) with confidence intervals, effect sizes, degrees of freedom and P value noted<br>*Give P values as exact values whenever suitable.*
√	☐	For Bayesian analysis, information on the choice of priors and Markov chain Monte Carlo settings
√	☐	For hierarchical and complex designs, identification of the appropriate level for tests and full reporting of outcomes
☐	√	Estimates of effect sizes (e.g. Cohen's d, Pearson's r), indicating how they were calculated

Our web collection on statistics for biologists contains articles on many of the points above.

Software and code

Policy information about availability of computer code

Data collection	Cobas c-311 Coulter chemistry analyzer, Illumina HiSeq X Ten (Illumina, San Diego, California, USA), Nikon fluorescence microscope (A1, Japan), Light Cycler 480 II apparatus (Roche, Germany), HT7700 transmission electron microscopy (Hitachi, Japan), UHPLC-Q/TOF-MS and Agilent Tapestation 2200 (Agilent technologies, USA), FACS Calibur flow cytometer (Cytoflex LX, China).
Data analysis	GraphPad Prism 8.0 analysis software, R (v4.0.0), Microsoft Excel 2010, ImageJ software (NIH, Bethesda, MD, USA), AlphaView SA software (ProteinSimple, San Jose, CA, USA), FlowJo V10 software. R Packages: CellRanger (v2.2.0), Seurat (v4.0.4), DoubletFinder (v2.0.3), Monocle (v2.1.6), Metascape (v3.5), clusterProfiler (v3.16.1), Cytoscape (v3.7.2), XCMS suite (v3.2), Bowtie2 (v2.2.6), DESeq2 (v1.28.1), MetaboAnalyst 5.0. The R code using for data processing is publicly available at GitHub at https://github.com/ZZlab412/Murine-thiamine-DATA/tree/V1.

For manuscripts utilizing custom algorithms or software that are central to the research but not yet described in published literature, software must be made available to editors and reviewers. We strongly encourage code deposition in a community repository (e.g. GitHub). See the Nature Portfolio guidelines for submitting code & software for further information.

Data

Policy information about availability of data

All manuscripts must include a data availability statement. This statement should provide the following information, where applicable:

- Accession codes, unique identifiers, or web links for publicly available datasets
- A description of any restrictions on data availability
- For clinical datasets or third party data, please ensure that the statement adheres to our policy

All relevant data are available within the article and its supplementary information/Source Data. The data of offspring ovarian single cell RNA sequencing, maternal gut microbiome, and offspring ovarian granulosa cells ATAC-sequencing that support the findings of this study has been deposited in the Genome Sequence Archive (GSA, https://ngdc.cncb.ac.cn/gsa) under the accession number CRA005316 (https://ngdc.cncb.ac.cn/gsa/browse/CRA005316), CRA005301 (https://ngdc.cncb.ac.cn/gsa/browse/CRA005301), and CRA005298 (https://ngdc.cncb.ac.cn/gsa/browse/CRA005298), respectively. Moreover, the data of maternal serum metabolomics have been deposited in the Open Archive for Miscellaneous Data (OMIX, https://ngdc.cncb.ac.cn/omix) under the accession number OMIX721 (https://ngdc.cncb.ac.cn/omix/release/OMIX721). Furthermore, in this study, the analysis was supplemented with the utilization of the KEGG (https://www.genome.jp/kegg) and GO (https://geneontology.org) databases for comprehensive functional enrichment analysis of the data. Source data are provided with this paper.

Research involving human participants, their data, or biological material

Policy information about studies with human participants or human data. See also policy information about sex, gender (identity/presentation), and sexual orientation and race, ethnicity and racism.

Reporting on sex and gender	Sex was considered in the study design. Women Study with healthy controls (HC) people and gestational diabetes mellitus (GDM) were recruited at Maternity and Child Health Hospital of Qingdao (Qingdao, China).
Reporting on race, ethnicity, or other socially relevant groupings	Chinese Han population
Population characteristics	Exclusion criteria comprised pre-existing diabetes, metabolic disorders, prior antibiotic use, history of alcohol or drug abuse, or the need for treatment for chronic diseases. Population characteristics are shown in Supplementary Table 1.
Recruitment	The follow-up participants were randomly chosen from pregnant women attending hospital appointments within one month, meeting inclusion criteria of around 26 weeks of gestation. No self-selection or other biases that might affect results exist.
Ethics oversight	The Medicine Ethics Committee of Qingdao Maternity and Child Health Hospital (IRB Number: [2021]074).

Note that full information on the approval of the study protocol must also be provided in the manuscript.

Field-specific reporting

Please select the one below that is the best fit for your research. If you are not sure, read the appropriate sections before making your selection.

☑ Life sciences ☐ Behavioural & social sciences ☐ Ecological, evolutionary & environmental sciences

For a reference copy of the document with all sections, see nature.com/documents/nr-reporting-summary-flat.pdf

Life sciences study design

All studies must disclose on these points even when the disclosure is negative.

Sample size	No specific statistical methods were used to predetermine the sample size. Sample sizes were chosen based on prior knowledge from previous experiments or preliminary data demonstrating statistically significant differences for each specific assay. For each experiment, the samples used were indicated in the figures or legends. The PubMed Identifier (PMID) for the relevant reference is: 33504491, 33351795, 33462408, 33725483, 32325354, 24995980, 29459681, 32755581.
Data exclusions	No data were excluded from the analyses.
Replication	Both in vitro and in vivo experiments were performed with at least 3 biological replicates.The numbers of replicates performed for each experiment are indicated in the Figure Legends. All results were reproducible.
Randomization	Random allocation.
Blinding	The investigators were blinded to group allocation during data collection and/or analysis.

Behavioural & social sciences study design

All studies must disclose on these points even when the disclosure is negative.

Study description	
Research sample	
Sampling strategy	
Data collection	
Timing	
Data exclusions	
Non-participation	
Randomization	

Ecological, evolutionary & environmental sciences study design

All studies must disclose on these points even when the disclosure is negative.

Study description	
Research sample	
Sampling strategy	
Data collection	
Timing and spatial scale	
Data exclusions	
Reproducibility	
Randomization	
Blinding	

Did the study involve field work? ☐ Yes ☐ No

Field work, collection and transport

Field conditions	
Location	
Access & import/export	
Disturbance	

Reporting for specific materials, systems and methods

We require information from authors about some types of materials, experimental systems and methods used in many studies. Here, indicate whether each material, system or method listed is relevant to your study. If you are not sure if a list item applies to your research, read the appropriate section before selecting a response.

Materials & experimental systems

n/a	Involved in the study	
☐	√	Antibodies
√	☐	Eukaryotic cell lines
√	☐	Palaeontology and archaeology
☐	√	Animals and other organisms
√	☐	Clinical data
√	☐	Dual use research of concern
√	☐	Plants

Methods

n/a	Involved in the study	
√	☐	ChIP-seq
√	☐	Flow cytometry
√	☐	MRI-based neuroimaging

Antibodies

Antibodies used

Details of antibodies are shown in Supplementary Table 2.

Validation

We declare that these antibodies have been validated, and their specificity has been confirmed through querying the manufacturer's official website and relevant literature. The detailed information about the antibodies can be found in Supplementary Table 2.DDX4 (IF/WB/IHC), Abcam (ab13840), https://www.abcam.com/products/primary-antibodies/ddx4--mvh-antibody-ab13840.html; DDX4 (IF), Abcam (ab27591), https://www.abcam.com/products/primary-antibodies/ddx4--mvh-antibody-mabcam27591-ab27591.html; LHX8 (IHC/WB), Abcam (ab137036), https://www.abcam.com/products/primary-antibodies/lhx8-antibody-epr52132-ab137036.html; NOBOX (IHC/WB), SANTA (sc-514178), https://www.scbt.com/p/nobox-antibody-a-5;α-tubulin (IF/WB), CST (3873), https://www.cellsignal.com/products/primary-antibodies/a-tubulin-dm1a-mouse-mab/3873; SLC19A3 (WB), Proteintech (13407-1-AP), https://www.ptglab.com/products/SLC19A3-Antibody-13407-1-AP.htm; GAPDH (WB), Proteintech (60004-1-IG), https://www.ptglab.com/products/GAPDH-Antibody-60004-1-Ig.htm; β-Actin (WB), CST (4967), https://www.cellsignal.com/products/primary-antibodies/b-actin-antibody/4967; PDH-E1 (WB/IF), CST (3205), https://www.cellsignal.com/products/primary-antibodies/pyruvate-dehydrogenase-c54g1-rabbit-mab/3205; H3 (WB), Proteintech (17168-1-AP), https://www.ptglab.com/products/Histone-H3-Antibody-17168-1-AP.htm; Ac-H3 (WB), Affinity (AF4365), https://www.affbiotech.cn/goods-14911-AF4365-Acetyl_Histone_H3_Lys9_14_18_23_27_Antibody.html; Ac-H3K9 (WB), CST (9649), https://www.cellsignal.com/products/primary-antibodies/acetyl-histone-h3-lys9-c5b11-rabbit-mab/9649; Ac-H3K18 (WB), CST (13998), https://www.cellsignal.com/products/primary-antibodies/acetyl-histone-h3-lys18-d8z5h-rabbit-mab/13998; H4 (WB), Proteintech (16047-1-AP), https://www.ptglab.com/products/HIST1H4E-Antibody-16047-1-AP.htm; Ac-H4 (WB), ActiveMotif (39026), https://www.activemotif.com/catalog/details/39925/histone-h4-pan-acetyl-antibody-pab-1; P-RB (WB), CST (8516), https://www.cellsignal.com/products/primary-antibodies/phospho-rb-ser807-811-d20b12-xp-rabbit-mab/8516; CDK4 (WB), Proteintech (11026-1-AP), https://www.ptglab.com/products/CDK4-Antibody-11026-1-AP.htm; CDK6 (WB), Proteintech (14052-1-AP), https://www.ptglab.com/products/CDK6-Antibody-14052-1-AP.htm; MCM6 (WB), Affinity (DF6716), https://www.affbiotech.cn/goods-5518-DF6716-MCM6_Antibody.html; CDK7 (WB), Affinity (DF6559), https://www.affbiotech.cn/goods-5361-DF6559-CDK7_Antibody.html; FOXL2 (IF), Abcam (ab5096), https://www.abcam.com/products/primary-antibodies/foxl2-antibody-ab5096.html.

Eukaryotic cell lines

Policy information about cell lines and Sex and Gender in Research

Cell line source(s)	
Authentication	
Mycoplasma contamination	
Commonly misidentified lines (See ICLAC register)	

Palaeontology and Archaeology

Specimen provenance	
Specimen deposition	
Dating methods	

☐ Tick this box to confirm that the raw and calibrated dates are available in the paper or in Supplementary Information.

Ethics oversight	

Note that full information on the approval of the study protocol must also be provided in the manuscript.

Animals and other research organisms

Policy information about studies involving animals; ARRIVE guidelines recommended for reporting animal research, and Sex and Gender in Research

Laboratory animals	7/8 weeks C57BL/6 and 8 weeks ICR female mice; 8 weeks C57BL/6 and 8 weeks ICR male mice.
Wild animals	The study did not involve wild animals.
Reporting on sex	Sex was considered in the study design, and female offspring were specifically investigated in relation to the impact of maternal high-fat diet during pregnancy.
Field-collected samples	This study did not contain samples derived from animals from the field.
Ethics oversight	All animal experiments were approved and performed in line with the Animal Care and Use Committee at Inner Mongolia University.

Note that full information on the approval of the study protocol must also be provided in the manuscript.

Clinical data

Policy information about clinical studies

All manuscripts should comply with the ICMJE guidelines for publication of clinical research and a completed CONSORT checklist must be included with all submissions.

Clinical trial registration	
Study protocol	
Data collection	
Outcomes	

Dual use research of concern

Policy information about dual use research of concern

Hazards

Could the accidental, deliberate or reckless misuse of agents or technologies generated in the work, or the application of information presented in the manuscript, pose a threat to:

No	Yes	
☐	☐	Public health
☐	☐	National security
☐	☐	Crops and/or livestock
☐	☐	Ecosystems
☐	☐	Any other significant area

Experiments of concern

Does the work involve any of these experiments of concern:

No	Yes	
☐	☐	Demonstrate how to render a vaccine ineffective
☐	☐	Confer resistance to therapeutically useful antibiotics or antiviral agents
☐	☐	Enhance the virulence of a pathogen or render a nonpathogen virulent
☐	☐	Increase transmissibility of a pathogen
☐	☐	Alter the host range of a pathogen
☐	☐	Enable evasion of diagnostic/detection modalities
☐	☐	Enable the weaponization of a biological agent or toxin
☐	☐	Any other potentially harmful combination of experiments and agents

Plants

Seed stocks

Novel plant genotypes

Authentication

ChIP-seq

Data deposition

☐ Confirm that both raw and final processed data have been deposited in a public database such as GEO.

☐ Confirm that you have deposited or provided access to graph files (e.g. BED files) for the called peaks.

Data access links
May remain private before publication.

Files in database submission

Genome browser session
(e.g. UCSC)

Methodology

Replicates

Sequencing depth

Antibodies

Peak calling parameters

Data quality

Software

Flow Cytometry

Plots

Confirm that:

- [ ] The axis labels state the marker and fluorochrome used (e.g. CD4-FITC).
- [ ] The axis scales are clearly visible. Include numbers along axes only for bottom left plot of group (a 'group' is an analysis of identical markers).
- [ ] All plots are contour plots with outliers or pseudocolor plots.
- [ ] A numerical value for number of cells or percentage (with statistics) is provided.

Methodology

Sample preparation

Instrument

Software

Cell population abundance

Gating strategy

- [ ] Tick this box to confirm that a figure exemplifying the gating strategy is provided in the Supplementary Information.

Magnetic resonance imaging

Experimental design

Design type

Design specifications

Behavioral performance measures

Imaging type(s)

Field strength

Sequence & imaging parameters

Area of acquisition

Diffusion MRI [ ] Used [ ] Not used

Preprocessing

Preprocessing software

Normalization

Normalization template

Noise and artifact removal

Volume censoring

Statistical modeling & inference

Model type and settings

Effect(s) tested

Specify type of analysis: [ ] Whole brain [ ] ROI-based [ ] Both

Statistic type for inference

(See Eklund et al. 2016)

Correction

Models & analysis

n/a	Involved in the study
☐	☐ Functional and/or effective connectivity
☐	☐ Graph analysis
☐	☐ Multivariate modeling or predictive analysis

Functional and/or effective connectivity

Graph analysis

Multivariate modeling and predictive analysis

This checklist template is licensed under a Creative Commons Attribution 4.0 International License, which permits use, sharing, adaptation, distribution and reproduction in any medium or format, as long as you give appropriate credit to the original author(s) and the source, provide a link to the Creative Commons license, and indicate if changes were made. The images or other third party material in this article are included in the article's Creative Commons license, unless indicated otherwise in a credit line to the material. If material is not included in the article's Creative Commons license and your intended use is not permitted by statutory regulation or exceeds the permitted use, you will need to obtain permission directly from the copyright holder. To view a copy of this license, visit http://creativecommons.org/licenses/by/4.0/
